# Prospective study of malaria in pregnancy, placental and congenital malaria in Northwest Colombia

**DOI:** 10.1186/s12936-024-04948-5

**Published:** 2024-04-25

**Authors:** Jaiberth Antonio Cardona-Arias, Jaime Carmona-Fonseca

**Affiliations:** 1 School of Microbiology, University of Antioquia UdeA, Medellín, Colombia; 2 School of Medicine, University of Antioquia UdeA., Research Group Coordinator “Salud y Comunidad-César Uribe Piedrahíta”, Medellín, Colombia

**Keywords:** Infectious complications of pregnancy, Malaria, Newborn, Placenta, Pregnancy

## Abstract

**Background:**

Pregnancy Associated Malaria (PAM) include malaria in pregnancy (MiP), placental malaria (PM), and congenital malaria (CM). The evidence available in Colombia on PAM focuses on one of the presentations (MiP, PM or CM), and no study longitudinally analyses the infection from the pregnant woman, passing through the placenta, until culminating in the newborn. This study determined the frequency of MiP, PM, and CM caused by *Plasmodium vivax, Plasmodium falciparum,* or mixed infections, according to Thick Blood Smear (TBS) and quantitative Polymerase Chain Reaction (qPCR). Identifying associated factors of PAM and clinical-epidemiological outcomes in northwestern Colombia.

**Methods:**

Prospective study of 431 pregnant women, their placenta, and newborns registered in the data bank of the research Group “Salud y Comunidad César Uribe Piedrahíta” which collected information between 2014 and 2020 in endemic municipalities of the departments of Córdoba and Antioquia. The frequency of infection was determined with 95% confidence intervals. Comparisons were made with the Chi-square test, Student t-test, prevalence ratios, and control for confounding variables by log-binomial regression.

**Results:**

The frequency of MiP was 22.3% (4.6% using TBS), PM 24.8% (1.4% using TBS), and CM 11.8% (0% using TBS). Using TBS predominated *P. vivax.* Using qPCR the proportions of *P. vivax* and *P. falciparum* were similar for MiP and PM, but *P. falciparum* predominated in CM. The frequency was higher in nulliparous, and women with previous malaria. The main clinical effects of PAM were anaemia, low birth weight, and abnormal APGAR score.

**Conclusions:**

The magnitude of infections was not detected with TBS because most cases were submicroscopic (TBS-negative, qPCR-positive). This confirmed the importance of improving the molecular detection of cases. PAM continue being underestimated in the country due to that in Colombia the control programme is based on TBS, despite its outcomes on maternal, and congenital health.

## Background

Pregnancy Associated Malaria (PAM) includes three presentations: (i) Malaria in Pregnancy (MiP), which corresponds to the presence of *Plasmodium* in the peripheral blood of a pregnant woman; (ii) Placental Malaria (PM), when infection of this organ is demonstrated; and (iii) Congenital Malaria (CM), or transplacental transmission of the parasites causing infection in the newborn, discarding bite of mosquitoes, or blood transfusion [1, 2].

PAM is related to severe outcomes, such as anaemia and death (maternal, fetal, and neonatal), intrauterine growth retardation, abortion, premature birth, and low birth weight [3–5]. Furthermore, in 2020, there were 248 million pregnancies worldwide, of which 157 million (63%) were registered in 85 malaria-endemic countries and 122 million (49%) occurred in areas with transmission of the parasite. In this last group, there were an estimated 1.4 million (1%) stillbirths, 33.5 million (27%) induced abortions, and 16.1 million (13%) spontaneous abortions [6].

These epidemiological consequences of MiP present high heterogeneity hinging on the level of endemicity, mother's immunity, social factors, and various lifestyles associated with the use of preventive methods and health services [3, 7]. There are also differences depending on the causal species; however, research on this topic has focused on *P. falciparum* for presenting greater mortality and morbidity [8], with few studies on the pathogenesis of *P. vivax*. For PAM due to *P. falciparum,* the cerebral malaria, anaemia, and respiratory distress have been related to its cytoadherence to infected erythrocytes [9]; specifically in PM, *P. falciparum-*infected erythrocytes adhere to placental receptors, causing placental inflammation and damage in pregnant women and newborns [8].

There are few studies in *P. vivax*, in which various pathological mechanisms such as erythrocyte sequestration and monocyte accumulation have been investigated. These studies have shown correlation of *P. vivax* with acute, sub-acute, and chronic PM, low fetal and birth weight [10]. Other authors have reported a higher prevalence of clinical manifestations in *P. vivax* infections, such as headache (81% Vs. 50% in *P. falciparum*), fever (73% Vs. 8% in *P. falciparum*), musculoskeletal pain (36% Vs. 4% in *P. falciparum*), and conjunctival pallor (12% Vs. 0% in *P. falciparum*), contrary to the evidence available in PAM that indicates greater damage by *P. falciparum* [11]. There are also important differences depending on the diagnostic test, evidencing important limitations of TBS for the diagnosis and epidemiological surveillance of PM and CM [12]. It has even been documented that in some contexts, the highest proportion of PAM infections are submicroscopic (positive with qPCR and negative with thick blood smear TBS), and they are associated with clinical outcomes [11] and constitute a great challenge for the elimination of malaria because the majority are asymptomatic [13, 14].

In Colombia, PAM is a topic with few epidemiological studies [15] and there are no official reports about this event because the malaria statistics of the Ministry of Health report the sex of the confirmed cases but do not indicate how many cases correspond to pregnant women. A meta-analysis of 14 studies determined the frequency of MiP, PM, and CM in 7932 pregnant women, 2506 placentas, 1143 umbilical cord samples, and 899 peripheral blood samples from neonates. Using TBS, the frequency of MiP was 5.8%, PM 3.4%, and CM 1.3%; using Polymerase Chain Reaction (PCR) the frequency of MiP was 16.7%, PM 11.0%, and CM 16.2%. Submicroscopic infections (negative with TBS and positive with PCR) were 8.5% in MiP, 10.1% in PM, and 22.0% in CM [16].

Some associated factors of PAM in Colombia include maternal and gestational age, number of pregnancies, parity, and previous malaria episodes; however, these factors were not consistent in research conducted in different regions of the country [16]. According to other studies the outcomes of PAM included gestational anaemia, severe malaria with liver dysfunction, acidosis or thrombocytopenia; alteration of the development of the immune response; abortion, premature birth, stillbirth, low birth weight, and lower height and head circumference at birth [3, 4, 6, 17–19].

The evidence available in Colombia on PAM focuses on one of the presentations (MiP, PM or CM), and no study longitudinally analyses the infection from the pregnant woman, passing through the placenta, until culminating in the newborn. This type of longitudinal analysis is decisive for the following reasons: (i) it allows evaluating the success of diagnosis, and treatment in pregnant women, follow-up and antenatal care programmes until birth, (ii) it demonstrates the importance of studying the complete clinical horizon (characteristics of the exposed population, development of MiP, placental involvement, infection in the newborn, and clinical outcomes), (iii) it quantifies the risk of PAM for maternal, fetal, and neonatal health, (iv) it identifies the most important species in each component of the trinomial (pregnant woman, placenta, newborn), as a reflection of the distribution of *P vivax* and *P. falciparum*, and the difference in the pathogenesis of each species involved in MiP, PM, and CM, and (v) it quantifies the cases of submicroscopic PAM, and the underreporting-underestimation that TBS generates, mainly in PM and CM.

Therefore, the objectives of this study were to determine the frequency of MiP, PM, and CM; and the specific frequency of PAM caused by *P. vivax*,* P. falciparum* or mixed infections, according to TBS, and qPCR; and to identified the associated factors, and clinical outcomes of PAM in northwestern Colombia.

## Methods

### Study population

Prospective study of pregnant women, their placentas and newborns, from in Northwest Colombia, a malaria-endemic region that includes 25 municipalities of two departments: Antioquia (Urabá and Bajo Cauca) and Córdoba (Sinú and San Jorge riverside); recruited during trimesters 2–3 of pregnancy in antenatal care appointments to performed diagnosis of GM, followed until the delivery to diagnose PM and CM.

The population consisted of 431 pregnancy women, placenta, and newborn trinomials, registered in the data bank of the Research Group “Salud y Comunidad César Uribe Piedrahíta” which was achieved with the collection of data between 2014 and 2020. This population met the following inclusion criteria: pregnant women, their placentas and newborns captured in the obstetric services of hospitals, stable residents of the study region, with realization of TBS and qPCR for malaria, and voluntary participation in the study. Exclusion criteria were: anti-malarial treatment in the previous two weeks to sample collection to perform TBS and qPCR, presence diseases or complications during pregnancy according to the evaluation the physician.

### Diagnosis of PAM

For GM diagnosis, a peripheral blood sample was taken from each pregnant woman by venipuncture. For PM one sample was taken for pathology in the insertion area of the umbilical cord, and another from the middle area of the placenta; from the space generated by the extraction of these tissues blood was taken to perform the diagnosis. For CM peripheral (heel) or cord blood samples were taken from each neonate on the day of delivery.

Using the same blood sample, slides were made for the microscopic diagnosis by TBS, and molecular diagnosis using qPCR with Whatman No.3 filter paper circles for DNA extraction with Saponin-Chelex method. In TBS 200 microscopic fields under 100X magnification were analyzed, this was positive when at least one parasitic form was observed, diagnosing infection by *P. falciparum*, *P. vivax*, or mixed-malaria (parasitic forms of *P. vivax* + gametocytes of *P. falciparum* or *P. vivax* +  ≥ 40% of regular asexual forms of *P. falciparum*). qPCR avoid post-amplification manipulation and quantify the number of microorganisms; it includes a highly conserved region 18S rRNA of *Plasmodium*, and primers polymorphic specific to *P. falciparum*, *P. vivax*, *Plasmodium malariae*, and *Plasmodium ovale* [20–22]

### Information gathering

The medical chart recorded the following data: age, health affiliation regime, trimester of pregnancy; number of pregnancies, deliveries, abortions, and stillbirths; use of insecticide-treated net, previous diagnosis of malaria, gestational anaemia (Haemoglobin < 11 g/dL), weight, height and head circumference at birth, and APGAR (Activity or muscle tone, Pulse, Grimace or reflex irritability, Appearance of the skin, and Respiration) score (normal with values ≥ 7). The clinical measurements of the pregnant women and their newborns were performed by the doctor who provided care to the pregnant woman.

In information bias control, there were training and standardizing the fieldwork, internal quality control in the laboratory, implementation of the test manufacturer's instructions, double and blind data entry. Confounding variables were analysed using generalized linear regression.

### Statistical analysis

The characteristics of the population were described with frequencies (*n* and %); it was determined the frequency of MiP, PM, and CM (general and by plasmodial species) with proportions and their 95% confidence intervals. Factors associated with PAM (positive for ≥ 1 of GM, PM, or CM) were identified using the Chi-square test, and Student t-test for continuous variables (weight, height and head circumference at birth). The assumption of normality was evaluated using Kolmogorov–Smirnov with correction of Lilliefors. In the variables that showed a statistical relationship with PAM, the association strength was determined using prevalence ratios and their 95% confidence interval. The confounding variables of PAM were identified using log-binomial regression. Analyses were performed using SPSS 29.0 with a significance < 0.05.

### Ethical aspects.

The ethical principles of the Declaration of Helsinki and Resolution 8430 of Colombia were applied. This study was classified as minimal risk and was endorsed by the Ethics Committee of the SIU (*in Spanish Sede de Investigación Univesitaria*), University of Antioquia, Minute 21-101-961. The participants signed the informed consent (of legal age) or assent (under 18 years of age), obtained in writing; it was also signed by a witness (external to the research group), and a member of the health team who explained its content. When it was possible, parental consent was obtained; however, according to rulings C-246/17 and T-675-17 on the self-determination of minors, the Constitutional Court of the Republic of Colombia in 2017 defined that parental consent is not necessary in these cases given that at 14 years of age, it has been established that minors may have the maturity to begin assuming obligations and responsibilities in society such as marriage, consenting to sexual relations, and the right to privacy in the family environment.

## Results

In the population, 94% were affiliated to the subsidized health regime (which groups unemployed people with no ability to pay economic contributions to health). The highest proportion of women were aged between 14 and 24 years (61.9%), in the last trimester of pregnancy (82.5%), multiparous (46.4%), with a high prevalence of anaemia (26.5%), abortions (14.2%), and newborns with low birth weight (6.5%) (Table 1).

**Table 1:** Description of the gynecological, obstetric, and neonatal characteristics

	n	%
Age group		
14–24 years	260	61.9
> 24 years	160	38.1
Trimester of pregnancy		
One and two (week 4–26)	73	17.5
Three (week 27–43)	343	82.5
Pregnancies		
One	131	31.2
Two	104	24.8
Three	77	18.3
Four or more	108	25.7
Deliveries		
Nulliparous (0)	91	28.7
Primiparous (1)	79	24.9
Multiparous (2 or more)	147	46.4
Other characteristics of the pregnant woman, and the newborn		
Use of insecticide-treated net	181	65.8
Diagnosis previous of malaria	98	22.7
Gestational anaemia	71	26.5
Abortions	38	14.2
Stillbirth	5	1.9
Low birth weight	26	6.5
Abnormal APGAR	3	1.2

In the variables there was missing data, therefore the % are not calculated on 431

**Fig. 1:**
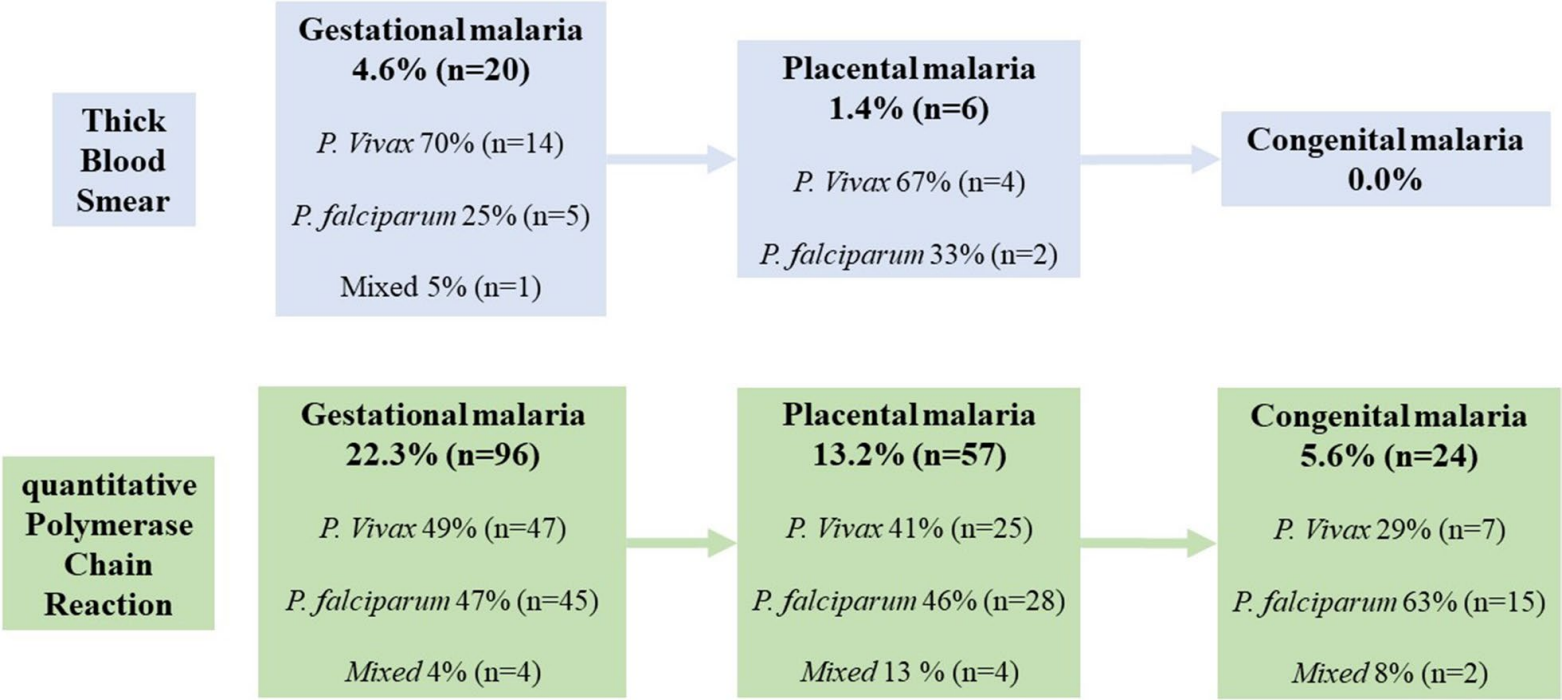
Follow-up the frequency of malaria in pregnant women, placentas and neonates using thick blood smear and quantitative polymerase chain reaction

Using TBS, the frequency of MiP was 4.6% and PM 1.4% with a predominance of *P. vivax;* without cases of CM. Using qPCR, the frequency of MiP was 22.3%, PM 13.2%, and CM 5.6%; likewise, the proportion per species changed, *P. vivax* and *P. falciparum* were similar in MiP and PM, while *P. falciparum* predominated in CM (Fig. 1).

PAM did not present statistical differences with age group, pregnancies, abortions, stillbirth, use of insecticide-treated net, height at birth, and head circumference at birth (Table 2).

**Table 2:** Bivariate analysis of pregnancy associated malaria (PAM) with factors that did not present association

Characteristics of the pregnant woman	Subgroups	PAM specific frequency %(*n*)^a^	*p*
Age group	14–24 years	31.5 (82)	0.077^b^
	> 24 years	40.0 (64)	
Pregnancies	One	29.8 (39)	0.418^b^
	Two	37.5 (39)	
	Three	40.3 (31)	
	≥ Four	36.1 (39)	
Use of insecticide-treated net	No	17.0 (16)	0.431^b^
	Yes	21.0 (38)	
Outcomes			
Abortions	No	20.1 (46)	0.812^b^
	Yes	18.4 (7)	
Stillbirth	No	19.9 (52)	0.997^b^
	Yes	20.0 (1)	

	Mean ± Standard deviation		
	Without PAM	With PAM	
Height at birth	49.8 ± 3.6	48.4 ± 5.3	0.085^c^
Head circumference at birth	33.8 ± 1.7	33.4 ± 2.2	0.145^c^

^a^Proportion of Pregnancy Associated Malaria PAM in the group of each row

^b^Chi squared

^c^t-Student Test

**Fig. 2:**
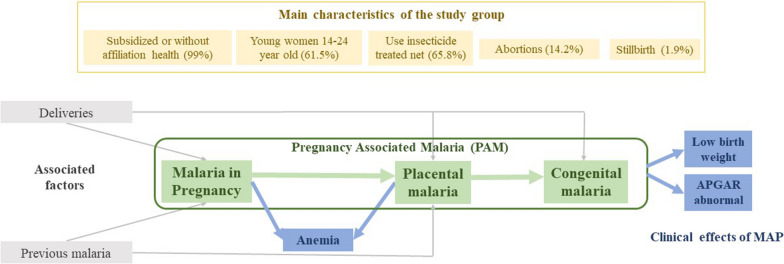
Synthesis of the main results

The associated factors with MAP were the number of deliveries, and previous malaria; PAM was twice higher in nulli/primiparous, and pregnant women with previous malaria diagnosis (Table 3). Gestational anaemia, low birth weight, and alteration of the APGAR score, were the main clinical outcomes of PAM (Table 4). Figure 2 shows the main findings of the study.

**Table 3:** Associated factors of pregnancy associated malaria

Variables and its categories	PAM specific frequency %(*n*)^a^	Crude prevalence ratio (95%CI)	Adjusted prevalence ratio (95%CI)
Trimester of pregnancy			
Three	37.6 (129)	1.61 (1.04–2.50)*	1.59 (0.79–3.22)
One and two	23.3 (17)		
Deliveries			
Multiparous (≥ 2)	15.6 (23)	1.00	1.00
Nulliparous (0)	31.9 (29)	2.03 (1.26–3.29)**	2.83(1.46–5.48)**
Primiparous (1)	27.8 (22)	1.78 (1.06–2.98)*	2.67(1.33–5.39)**
Previous malaria			
Yes	55.1 (54)	1.89 (1.48–2.42)**	2.26 (1.17–4.37)*
No	29.1 (97)		

^a^ Proportion of Pregnancy Associated Malaria (PAM) in the group of each row

*p < 0.05. **p < 0.01

**Table 4:** Outcomes associated with pregnancy associated malaria (PAM)

Outcomes	With PAM %(n)^a^	Without PAM %(n)^a^	Crude prevalence ratio (95%CI)	Adjusted prevalence ratio (95%CI)
Gestational anaemia	40.4 (46)	16.2 (25)	2.48 (1.63–3.79)**	3.36 (1.87–6.03)**
Low birth weight	11.0 (16)	3.9 (10)	2.79 (1.30–6.00)**	4.77 (1.49–15.27)**
Abnormal APGAR	6.3 (3)	0.0 (0)	NA	NA

^a^Proportion of each outcome (the denominator was each group with or without PAM)

**p < 0.01

## Discussion

The characteristics of the population studied show that PAM is concentrated in women without monetary resources (health affiliation is a *proxy* of the economic situation); thus, control of PAM should be concomitant with interventions of the economic dimension as has been shown in other studies [23, 24]. The coexistence of MiP, PM, and CM shows the importance of diagnosing, monitoring, and treating the three forms of PAM, avoiding the consequences that have been documented in other studies such as anaemia, severe malaria, and death in the mother; anaemia, and death fetal; abortion, stillbirth, low birth weight, and CM [3–5].

The frequency of MiP was 22.3% (79% of the cases were submicroscopic); PM was 24.8% (97% of the cases were submicroscopic), and CM 11.8% (100% of the cases were submicroscopic). These data coincide with a meta-analysis that grouped the available evidence in Colombia, showing that the highest proportion of PAM cases are submicroscopic [16]. Considering that TBS is the diagnostic test used in Colombia the current epidemiological surveillance, and infection control actions in the country have been classified as a program with a good impact, but true risk of MiP is not being captured, much less in PM, and CM, where almost all cases are submicroscopic. This situation is serious considering that submicroscopic cases do not allow transmission to be avoided or stopped; and these cases can progress to the clinical effects on maternal, fetal and neonatal health [25–27].

In parasitological terms, with TBS the proportion of *P. vivax* was higher, whereas with qPCR the proportion of *P. vivax* and *P. falciparum* were similar in MiP and PM, but *P. falciparum* predominated in CM; similar to other Colombian studies [16]. This demonstrates that TBS not only underestimates PAM, but also generates an epidemiological and parasitological profile with detection biases.

Finding a similar proportion of *P. vivax* and *P. falciparum* in MiP and PM, and both associated with gestational anaemia, contradicts several assumptions about the lower pathogenicity of *P. vivax* [28]. This evidence is added to a synthesis of the available evidence that reported serious effects of *P. vivax* infection such as anaemia, haematological complications, severe malaria, acute respiratory distress, multiple organ failure, and other pathological effects [28–31]. In addition, it is important to highlight that the higher proportion of cases of CM due to *P. falciparum* is a reflection of greater virulence, and possibilities of crossing the placenta [32].

The associated factors with PAM were the number of deliveries, and previous malaria in current pregnancy, which differs from other associated factors reported in Colombian studies such as maternal and gestational age, and number of pregnancies, but it coincides with parity, and previous malaria [16]. For example, a study found that 75% of pregnant women with MiP reported a previous episode, and 25% reported 2–4 episodes [3]. This shows several issues.

i. The investigation of risk factors for PAM in Colombia is incipient, and the few available studies are not exhaustive in the factors studied due to structural problems in the country's health information systems.
ii. Multiparity is a protective factor for PAM, therefore subsequent studies should broaden the understanding of immune mechanisms underlying this effect, as was suggested by other authors who reported that pathology of MiP is dependent on cytokine imbalances, placental sequestration, and parity [33].
iii. Programmes for antenatal care, epidemiological surveillance, and public health in general, should prioritize diagnosis and health education-communication actions for nulliparous, and primiparous women, given their greater risk of malaria.
iv. Finding a higher frequency of PAM in women with previous episodes of malaria shows at least three aspects that require further investigation; first, low effectiveness of epidemiological surveillance, care, and prevention programs; second, the need to study individual determinants of PAM prevention; and third, the ignorance of the social, economic, political and cultural determinants of malaria that would explain the persistence of the disease in these women and their children.

The main clinical effects of PAM were gestational anaemia, and low birth weight similar to previous studies conducted in Colombia [3, 4, 6, 17–19]. Abnormal APGAR scores have been poorly analysed in PAM, therefore it is not possible to contrast this result. This demonstrates the serious clinical effects of PAM, although antenatal care medical records should improve the completeness of the clinical history, to improve the evidence about other PAM outcomes that not yet investigated in Colombia.

### Limitations and recommendations

The medical chart does not allow a rigorous investigation of the factors associated with PAM, and its clinical outcomes. There are difficulties in accessing to some endemic territories because of being remote, or having armed conflict. Several women did not accept participate in the study, and they did not explain the reason for their refusal; based on a previously published qualitative study [34] the main reason to refuse is that they live very far from the hospital, and they haven’t time for attend their health topics. In other cases, health workers who attended the birth forgot to take samples for the diagnosis of placental malaria.

Despite these limitations, this research makes it possible to enunciate several recommendations: increase the frequency of PAM screening in endemic areas (minimum once a month), realize active search of cases of MiP (in their homes and workplaces), improve the data recording systems in the medical chart, and deepen the research on submicroscopic PAM and its clinical effects.

## Conclusion

The region presents a high frequency of MiP, PM and CM; which shows shortcomings in the treatment, control, and follow-up of cases. The frequency was higher in nulliparous, and women with previous malaria, making it possible to prioritize groups with the highest occurrence of the infection. The magnitude of infections and their risk factors were not detected with TBS, because almost all cases were submicroscopic, which confirms the importance of improving the detection of cases with other tests. This epidemiological profile showed serious clinical consequences of PAM for maternal, fetal, newborn, and child health.

## Data Availability

All relevant data supporting the conclusions of this article are included within the article. Any additional information is available from the corresponding author upon reasonable request.

## Abbreviations

PAM: Pregnancy associated malaria
MiP: Malaria in pregnancy
PM: Placental malaria
CM: Congenital malaria
qPCR: Quantitative polymerase chain reaction
TBS: Thick blood smear
